# High-Density Culture and Lipopolysaccharide Cooperatively Activate TAK1–MAPK Signaling to Promote Osteogenic Differentiation in Human Dental Pulp Stem Cells

**DOI:** 10.3390/ijms27219515

**Published:** 2026-10-24

**Authors:** Khaing Nyein Pwint, Nobuyuki Kawashima, Yamato Okada, Sonoko Noda, Takashi Okiji, Yoshio Yahata

**Affiliations:** 1 Department of Pulp Biology and Endodontics, Graduate School of Medical and Dental Sciences, Institute of Science Tokyo, Tokyo 113-8549, Japan; knp.endo@tmd.ac.jp (K.N.P.); y.okada.endo@tmd.ac.jp (Y.O.); s.noda.endo@gmail.com (S.N.); t.okiji@tky.ndu.ac.jp (T.O.); yahata.y.b157@m.isct.ac.jp (Y.Y.); 2 Department of Endodontics, School of Life Dentistry at Tokyo, The Nippon Dental University, Tokyo 102-8159, Japan

**Keywords:** human dental pulp stem cell, high-density culture, lipopolysaccharide, osteogenic differentiation, TAK1–MAPK signaling

## Abstract

Human dental pulp stem cells (hDPSCs) are promising for regenerative medicine because of their multipotent differentiation capacity and ease of isolation from extracted teeth. We previously reported that high-density culture enhanced their osteogenic potential; however, the inflammatory environment at the transplantation site may influence osteogenic differentiation. This study investigated the effects of lipopolysaccharide (LPS) on the osteogenic differentiation of hDPSCs cultured at high density. hDPSCs were seeded at either low or high density and stimulated with LPS. High-density culture increased focal adhesion kinase (FAK) phosphorylation, a well-established marker of integrin signaling activation, and enhanced osteogenic differentiation. Under high-density culture conditions, LPS further increased the expression of the osteogenic markers bone morphogenetic protein 2 (*BMP2*) and runt-related transcription factor 2 (*RUNX2*) and promoted mineralized nodule formation. Moreover, LPS stimulation induced the phosphorylation of transforming growth factor-β-activated kinase-1 (TAK1), p38 and c-Jun N-terminal kinase (JNK) exclusively under high-density culture conditions. TAK1 inhibition suppressed the phosphorylation of p38 and JNK and reduced *BMP2* and *RUNX2* expression. TAK1 phosphorylation was attenuated by a FAK inhibitor in LPS-stimulated hDPSCs cultured at high density. These findings suggest that FAK signaling associated with high-density culture facilitates LPS-induced TAK1–MAPK activation, contributing to increased *BMP2* and *RUNX2* expression and enhanced mineralized nodule formation in hDPSCs.

## 1. Introduction

Human dental pulp stem cells (hDPSCs) are mesenchymal stem cells present in the neural crest-derived dental pulp tissues [1]. They possess broad multi-differentiation potential and robust regenerative capacity [2], giving rise to various specialized cell types, including odontoblasts, osteoblasts, chondrocytes, neural cells, and adipocytes, under appropriate inductive conditions [3,4,5,6]. Their accessibility from extracted teeth, including third molars, provides a minimally invasive autologous stem cell source. Moreover, hDPSCs exhibit higher proliferative capacity and shorter population doubling time than human bone marrow mesenchymal stem cells (BMMSCs) while maintaining comparable osteogenic potential [7]. These characteristics have positioned hDPSCs as a promising and clinically relevant alternative to BMMSCs for applications in various types of tissue regeneration.

Among the factors that regulate the behavior of hDPSCs, in vitro cell culture conditions, particularly cell density, play a decisive role [8]. In hDPSCs cultured at high density, osteogenic marker expression and osteogenic differentiation capacity are markedly enhanced compared with those in low-density cultures [9]. Increased cell density enhances tight cell–cell contact formation, which further activate integrin-mediated signaling pathways such as focal adhesion kinase (FAK) and downstream phosphoinositide 3-kinase (PI3K) [9]. This signaling cascade promotes alkaline phosphatase, biomineralization associated (*ALPL*) mRNA expression, thereby facilitating osteoblastic commitment [9]. Consistent with these molecular findings, hDPSCs cultured at high density exhibit superior bone-forming ability in vivo compared with those cultured at low density [9].

In addition to physical culture conditions, inflammatory signals can modulate the behavior and differentiation potential of hDPSCs in a context-dependent manner [10]. Lipopolysaccharide (LPS), a major component of the outer membrane of Gram-negative bacteria such as *Escherichia coli*, is widely used to model the in vitro inflammatory microenvironment [11]. In hDPSCs, LPS activates Toll-like receptor 4 (TLR4) signaling, leading to cytokine production and the activation of downstream pathways that modulate cell behavior [12,13]. However, the impact of LPS on the osteo/odontogenic differentiation of hDPSCs remains controversial [14]. Some studies have reported that LPS-induced inflammation impairs the differentiation potential of hDPSCs [15,16], whereas others have demonstrated enhanced odontogenic differentiation, characterized by increased expression of dentin/bone matrix proteins and mineralized nodule formation, via the mitogen-activated protein kinase (MAPK) signaling pathway [17,18]. Since high-density culture promotes integrin–FAK signaling, which contributes to osteogenic differentiation [9], this cellular context may also influence the response of hDPSCs to inflammatory cues. However, the role of high-density culture-associated integrin–FAK signaling in LPS-induced osteogenic differentiation remains unclear.

Transforming growth factor-β-activated kinase-1 (TAK1), also known as MAP3K7, is a central mediator linking inflammatory and differentiation signals. TAK1, a serine/threonine kinase, is activated by diverse stimuli, including inflammatory cytokines, TLR ligands such as LPS, and mechanical stress, and subsequently regulates MAPK and nuclear factor-kappa B (NF-κB) pathways [19]. These pathways orchestrate key biological processes, including innate immunity, inflammatory response, cell proliferation, and differentiation [20,21]. In mesenchymal stem cells, TAK1 serves as a pivotal regulator of osteogenic differentiation, principally via the stimulation of MAPK signaling and the control of essential transcription factors such as runt-related transcription factor 2 (*RUNX2*) [22]. Importantly, TAK1 represents a potential convergence point for integrin-mediated mechanotransduction and TLR4-mediated inflammatory signaling; however, the interaction between these pathways remains poorly understood.

This study investigated how LPS stimulation modulates the osteogenic differentiation of hDPSCs cultured at high density, with particular emphasis on the underlying mechanisms mediated by TAK1-dependent signaling.

## 2. Results

### 2.1. LPS Stimulation in hDPSCs Cultured at High Density Alters the Transcriptomic Profile and Enhances Osteogenic Differentiation

**Figure 1:**
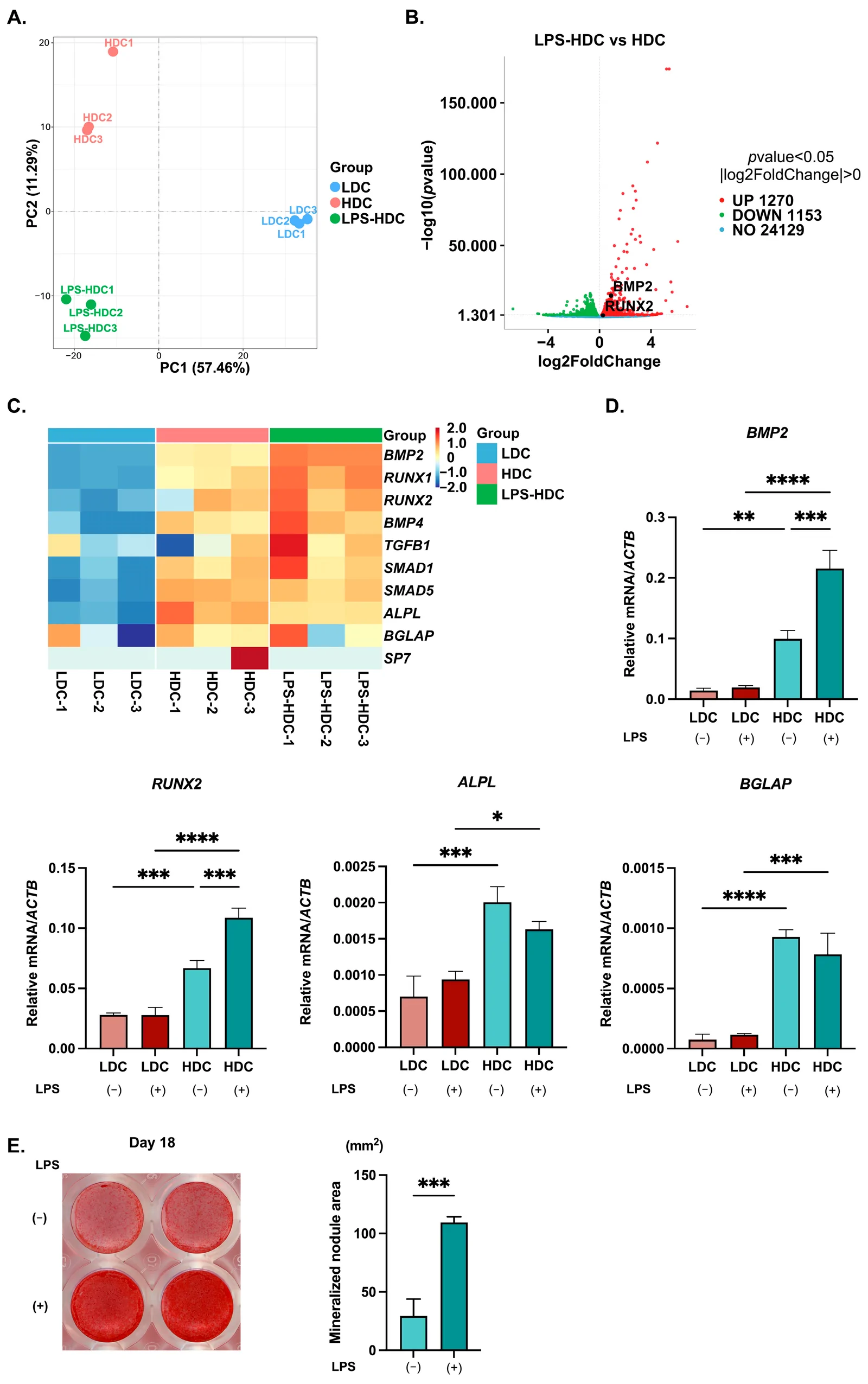
Effects of high-density culture and LPS stimulation on the transcriptomic profile and osteogenic differentiation of hDPSCs. (**A**) Principal component analysis showing distinct global gene expression profiles among the experimental groups. (**B**) Volcano plot of differentially expressed genes in hDPSCs cultured at high density with or without LPS stimulation, with significantly upregulated (red), significantly downregulated (green), and non-significant (blue) genes (*p* < 0.05). (**C**) Heatmap showing the expression pattern of osteogenesis-related genes in three experimental groups. Gene expression levels are represented as row-wise Z-scores, with red indicating relatively high expression and blue indicating relatively low expression. (**D**) hDPSCs cultured under high-density conditions for 3 days showed increased mRNA expression of osteogenic markers. LPS treatment (100 ng/mL) for 48 h further increased *BMP2* and *RUNX2* mRNA expression in high-density cultures, whereas no significant upregulation was observed in low-density cultures (mean ± SD, *n* = 3). (**E**) Alizarin Red S staining. Mineralized nodule formation was induced in hDPSCs cultured in the osteogenic medium with or without LPS for 18 days. Nodule formation was accelerated by the addition of LPS (mean ± SD, *n* = 4). * *p* < 0.05; ** *p* < 0.01; *** *p* < 0.001; **** *p* < 0.0001. LPS: lipopolysaccharide; hDPSC: human dental pulp stem cell; LDC: low-density culture; HDC: high-density culture; LPS-HDC: LPS-stimulated high-density culture.

Principal component analysis of RNA-sequencing data demonstrated clear separation between three experimental groups, indicating distinct global gene expression profiles (Figure 1A). Differential expression analysis between hDPSCs cultured at high density with or without LPS stimulation identified 1270 upregulated and 1153 downregulated genes (Figure 1B). Heatmap analysis demonstrated a progressive increase in the expression of osteogenesis-related genes, including bone morphogenetic protein 2 (*BMP2*), runt-related transcription factor 1 (*RUNX1*), *RUNX2*, and bone morphogenetic protein 4 (*BMP4*), from low-density hDPSCs to high-density hDPSCs, and further to LPS-stimulated high-density hDPSCs (Figure 1C). Furthermore, reverse-transcription quantitative polymerase chain reaction (RT-qPCR) was performed to validate the expression of osteogenic marker genes. hDPSCs cultured at high density showed significantly higher expression of *BMP2*, *RUNX2*, *ALPL*, and bone gamma-carboxyglutamate protein (*BGLAP*) than those at low density. Moreover, LPS stimulation further enhanced *BMP2* and *RUNX2* mRNA expression only under high-density conditions, whereas no significant further upregulation was observed for *ALPL* and *BGLAP*. In contrast, LPS stimulation induced no significant increase in the expression of any of the examined osteogenesis-related genes under low-density conditions (Figure 1D). To investigate the effects of LPS stimulation on osteogenic differentiation under high-density culture conditions, we also performed Alizarin Red S staining to assess mineralized nodule formation in hDPSCs cultured in osteogenic medium. LPS-treated cells exhibited a significant increase in mineralized nodule formation compared with untreated controls (Figure 1E).

### 2.2. Enhanced Phosphorylation of FAK and ERK in High-Density Culture Conditions

**Figure 2:**
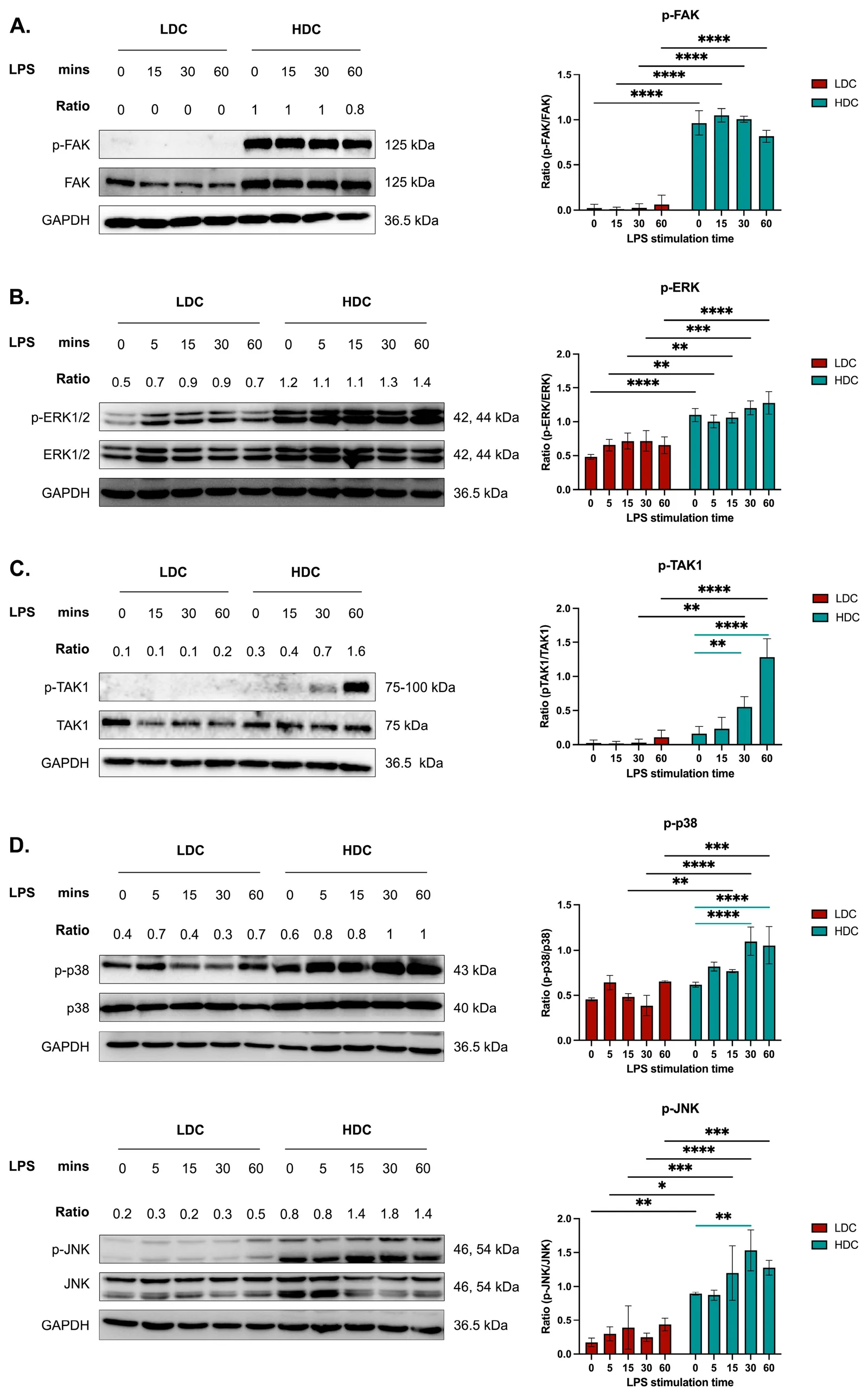
Time-course analysis of FAK, ERK, TAK1, p38 and JNK phosphorylation in hDPSCs following LPS stimulation. hDPSCs were cultured under low- or high-density conditions for 3 days and stimulated with LPS (100 ng/mL) for 5, 15, 30, or 60 min. (**A**,**B**) Phosphorylation levels of FAK and ERK were increased in hDPSCs cultured at high density. LPS stimulation did not alter the phosphorylation of FAK and ERK in hDPSCs cultured at high density. (**C**) LPS stimulation induced the phosphorylation of TAK1 in hDPSCs cultured at high density in a time-dependent manner. (**D**) LPS stimulation upregulated the phosphorylation of p38 and JNK in hDPSCs cultured at high density. GAPDH was used as a loading control. The ratios above the blots represent the levels of each phosphorylated protein relative to its corresponding total protein in the representative experiment. Bar charts are presented as mean ± SD (*n* = 3). Black horizontal lines indicate comparisons between the LDC and HDC groups, whereas blue horizontal lines indicate comparisons between different LPS stimulation time points and the untreated control (0 min) within the HDC group. * *p* < 0.05; ** *p* < 0.01; *** *p* < 0.001; **** *p* < 0.0001. LPS: lipopolysaccharide; hDPSC: human dental pulp stem cell; LDC: low-density culture; HDC: high-density culture.

To confirm the involvement of integrin signaling in high-density culture conditions, we examined the phosphorylation of FAK and its downstream factor, extracellular signal-regulated kinase (ERK), by Western blotting. FAK phosphorylation was markedly increased in hDPSCs cultured at high density, independent of LPS stimulation (Figure 2A). Similarly, ERK phosphorylation was elevated under these conditions, with minimal additional effect from LPS (Figure 2B).

### 2.3. LPS-Induced Activation of TAK1 and MAPK Signaling Pathway in hDPSCs

To investigate the involvement of TAK1 and its downstream MAPK signaling pathway in response to LPS, we analyzed total and phosphorylated forms of TAK1, p38, and c-Jun N-terminal kinase (JNK). Phosphorylation of all three proteins was elevated under high-density culture conditions. Upon LPS stimulation, phosphorylation levels of TAK1, p38, and JNK further increased (Figure 2C,D).

### 2.4. Effect of TAK1 Inhibitors on Phosphorylation of TAK1 and MAPK Signaling Components

**Figure 3:**
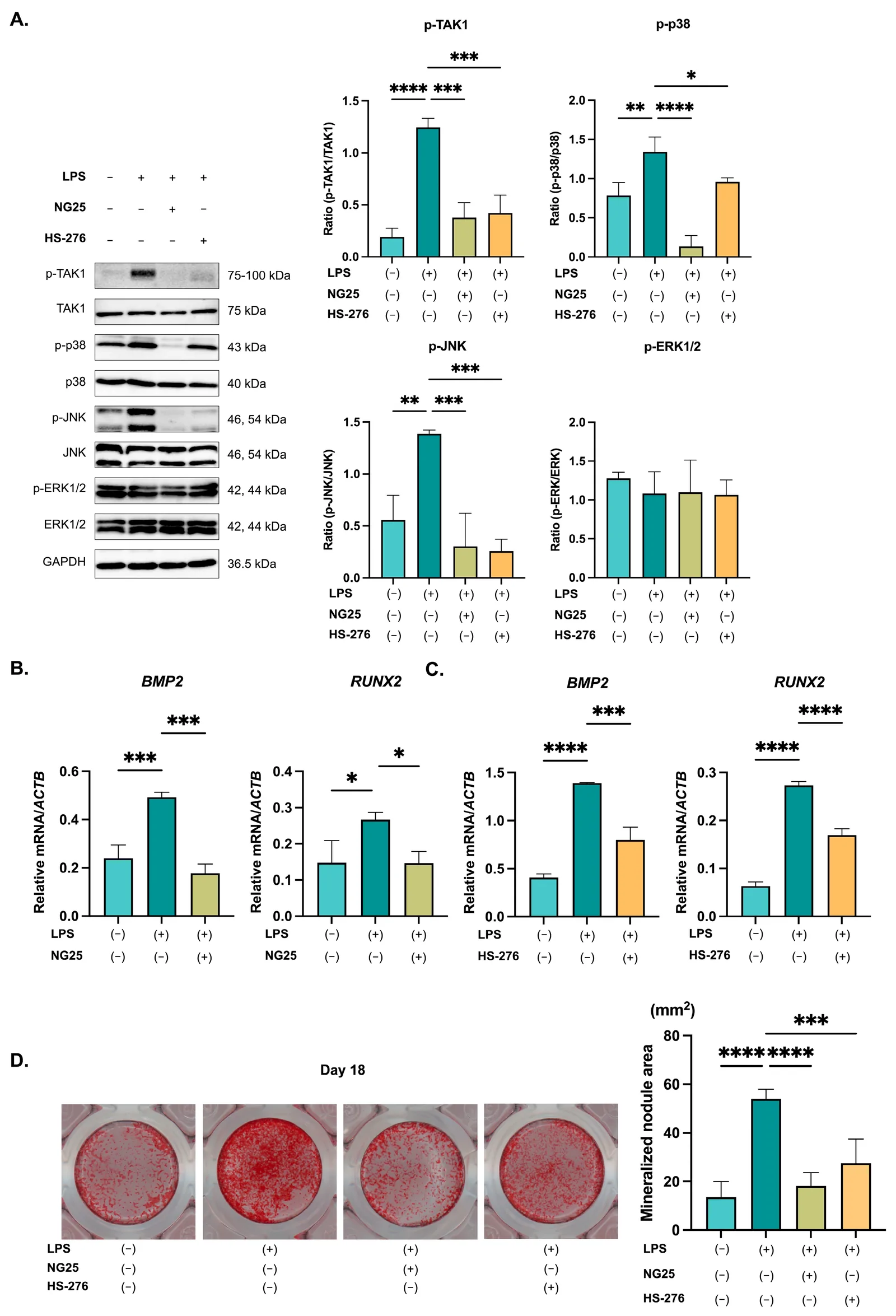
Effects of NG25 and HS-276 on LPS-induced TAK1 and MAPK pathway phosphorylation and osteogenic differentiation in high-density hDPSCs. hDPSCs cultured at high density were pretreated with NG25 (5 μM) or HS-276 (4 μM) 1 h prior to LPS treatment. For Western blot analysis, hDPSCs were cultured under high-density conditions for 3 days and stimulated with LPS for 1 h. (**A**) LPS stimulation significantly increased the phosphorylation of TAK1, p38, and JNK in hDPSCs cultured at high density, which was significantly downregulated by NG25 and HS-276 (mean ± SD, *n* = 3). For osteogenic gene expression analysis, hDPSCs were cultured under high-density conditions for 3 days and stimulated with LPS for 48 h. NG25 (**B**) and HS-276 (**C**) significantly downregulated *BMP2* and *RUNX2* mRNA expression in hDPSCs, which was upregulated by LPS stimulation in high-density culture conditions (mean ± SD, *n* = 3). (**D**) For mineralized nodule formation analysis, hDPSCs were cultured in osteogenic medium for 18 days. An equivalent volume of DMSO was added to samples not treated with the inhibitors as a vehicle control. Both NG25 and HS-276 significantly attenuated the LPS-enhanced mineralized nodule formation (mean ± SD, *n* = 4). * *p* < 0.05; ** *p* < 0.01; *** *p* < 0.001; **** *p* < 0.0001. LPS: lipopolysaccharide; hDPSC: human dental pulp stem cell.

LPS-stimulated hDPSCs cultured at high density were treated with TAK1 inhibitors (NG25 and HS-276), after which the phosphorylation of TAK1, p38, JNK, and ERK was examined. LPS stimulation significantly increased the phosphorylation of TAK1, p38, and JNK. Both NG25 and HS-276 effectively suppressed the LPS-induced phosphorylation of TAK1, p38, and JNK in hDPSCs cultured at high density (Figure 3A). In contrast, ERK phosphorylation remained unchanged upon either LPS treatment or TAK1 inhibition (Figure 3A).

### 2.5. Effect of TAK1 Inhibitors on Osteogenic Differentiation of hDPSCs

To determine whether TAK1 signaling contributes to LPS-induced osteogenic differentiation in hDPSCs cultured at high density, cells were pretreated with NG25 and HS-276 1 h before LPS stimulation. Both inhibitors significantly reduced the expression of *BMP2* and *RUNX2*, which were upregulated in high-density conditions following LPS stimulation (Figure 3B,C). In addition, LPS-enhanced mineralized nodule formation was significantly reduced by treatment with the inhibitors (Figure 3D). These findings indicate that TAK1 activity is required for the LPS-mediated induction of osteogenic differentiation in hDPSCs cultured at high density.

### 2.6. Effect of Upstream Pathway Inhibitors on TAK1 Phosphorylation

**Figure 4:**
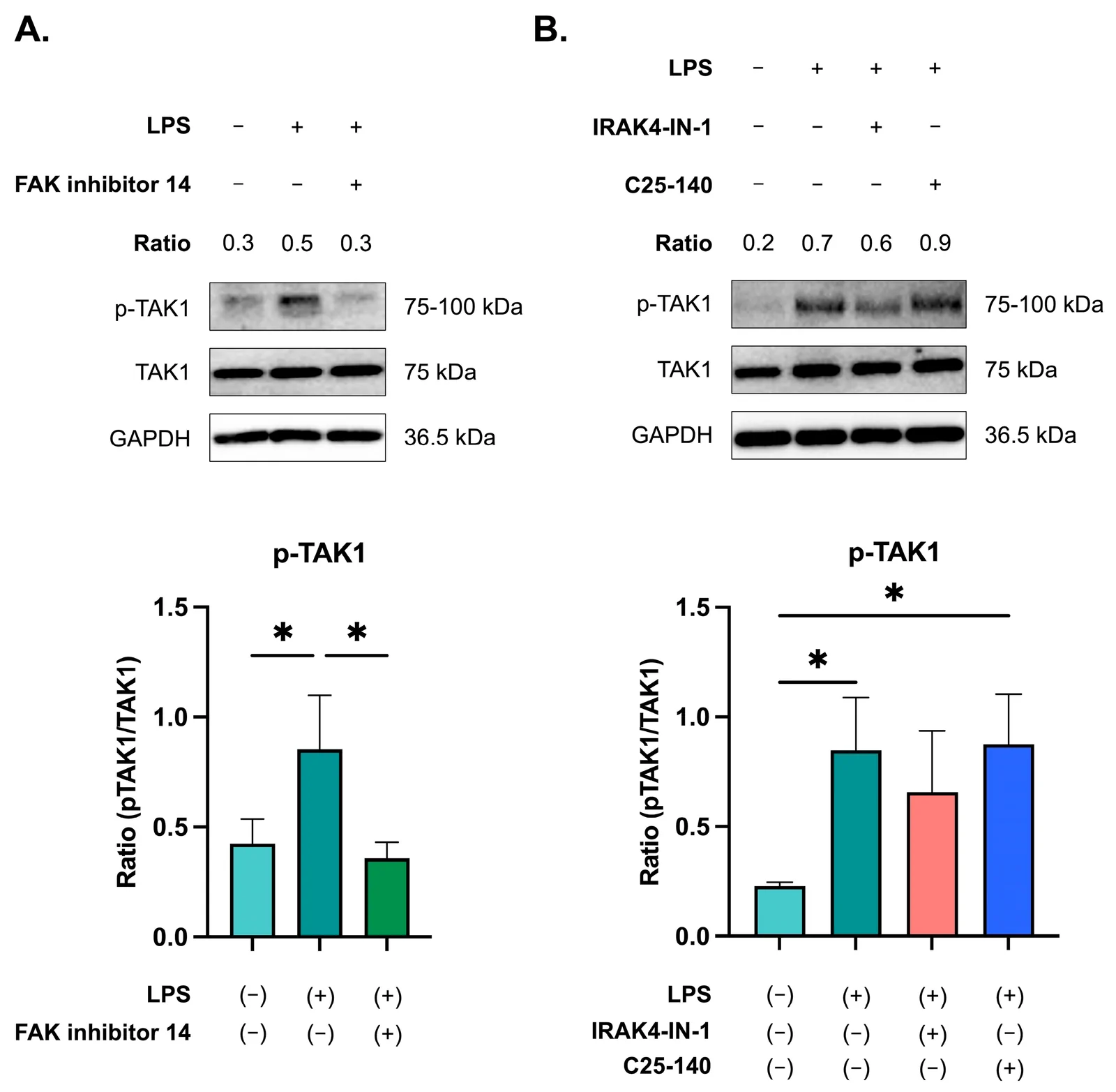
Effects of FAK, IRAK4, and TRAF6 inhibitors on TAK1 phosphorylation. hDPSCs were cultured under high-density conditions for 3 days and pretreated with the indicated inhibitors for 1 h prior to LPS stimulation. Western blot analysis of phosphorylated TAK1 and total TAK1 was performed after LPS stimulation for 1 h. (**A**) Treatment with FAK inhibitor 14 (1 μM) attenuated TAK1 phosphorylation (mean ± SD, *n* = 3). (**B**) IRAK4 inhibition with IRAK4-IN-1 (10 μM) slightly downregulated TAK1 phosphorylation; however, C25-140 (20 μM) did not suppress TAK1 phosphorylation (mean ± SD, *n* = 3). * *p* < 0.05. LPS: lipopolysaccharide; hDPSC: human dental pulp stem cell.

To investigate upstream regulators of TAK1 activation in the LPS-stimulated hDPSCs cultured at high density, pharmacological inhibitors targeting key signaling pathways were applied, and TAK1 phosphorylation was evaluated. Inhibition of FAK markedly reduced TAK1 phosphorylation, which was otherwise elevated upon LPS stimulation (Figure 4A). In contrast, the inhibition of interleukin-1 receptor-associated kinase 4 (IRAK4) with IRAK4-IN-1 resulted in only a slight reduction in TAK1 phosphorylation, and the inhibition of TNF receptor-associated factor 6 (TRAF6) using C25-140 did not suppress TAK1 phosphorylation (Figure 4B).

### 2.7. Activation and Role of NF-κB Signaling in LPS-Induced Osteogenic Differentiation of hDPSCs

**Figure 5:**
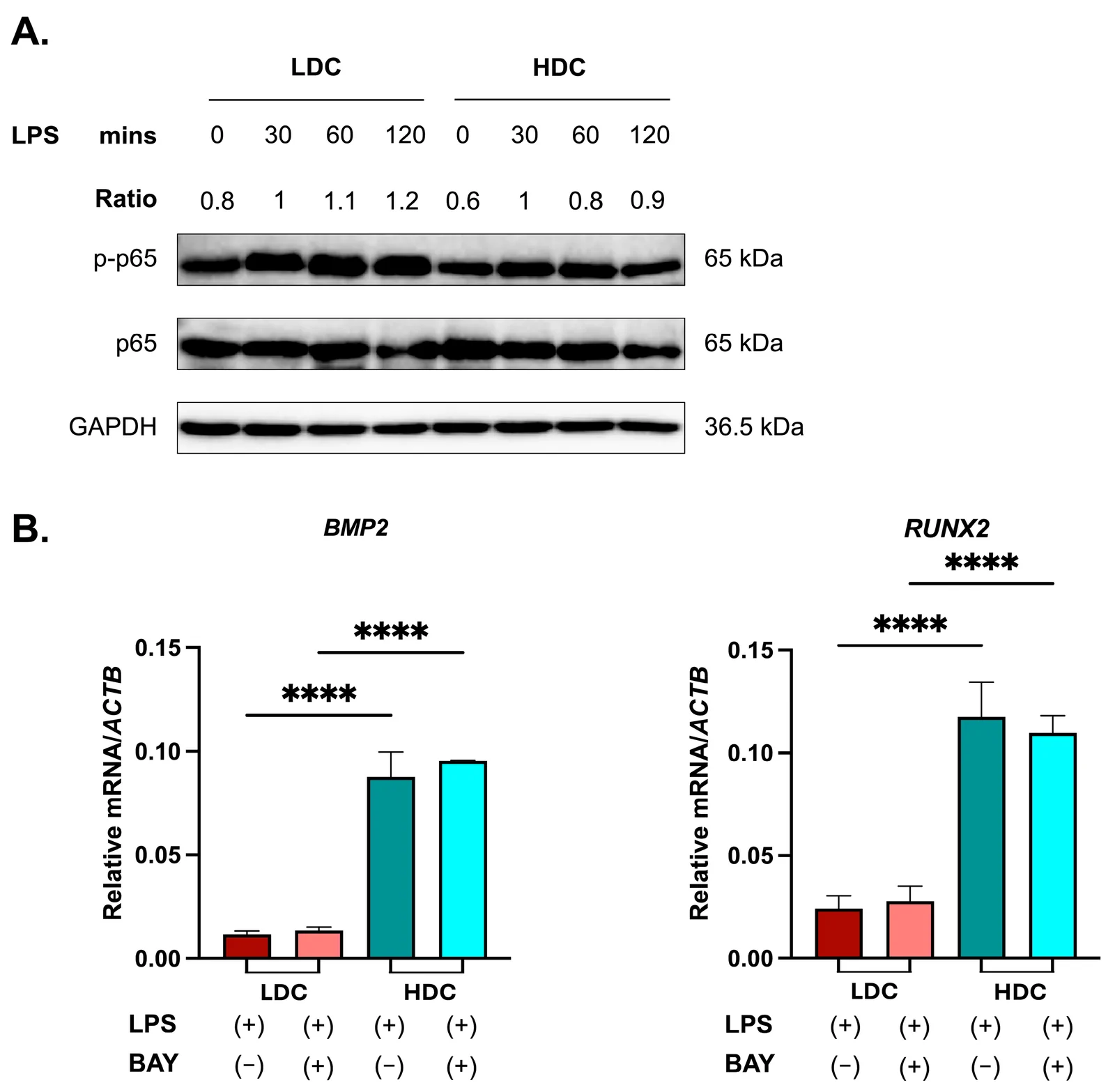
NF-κB signaling in hDPSCs under LPS stimulation. hDPSCs were cultured under low- or high-density conditions for 3 days. For Western blot analysis, cells were stimulated with LPS (100 ng/mL) for 30, 60, or 120 min. (**A**) LPS treatment increased the phosphorylation of p65 in hDPSCs cultured at both low and high density. For osteogenic gene expression analysis, cells were pretreated with the inhibitor for 1 h prior to LPS stimulation and then cultured with LPS for 48 h. (**B**) BAY 11-7085 (NF-κB inhibitor, 1 μM) did not alter osteogenic marker expression in LPS-stimulated hDPSCs (mean ± SD, *n* = 3). **** *p* < 0.0001. LPS: lipopolysaccharide; hDPSC: human dental pulp stem cell; LDC: low-density culture; HDC: high-density culture; BAY: BAY 11-7085.

Next, we examined whether NF-κB signaling, another downstream pathway of TAK1 in addition to MAPK signaling, is involved in LPS-induced osteogenic differentiation. Kyoto Encyclopedia of Genes and Genomes (KEGG) pathway enrichment analysis demonstrated significant enrichment of the NF-κB signaling pathway in LPS-stimulated high-density hDPSCs (Supplementary Figure S1), suggesting activation of NF-κB signaling. Western blot analysis also revealed the upregulation of p65 phosphorylation in LPS-stimulated low- and high-density cultures, indicating activation of the NF-κB pathway (Figure 5A). These findings indicated that LPS activates inflammation-related signaling under both density conditions and that activation of inflammatory signaling and enhancement of osteogenic differentiation occur concurrently in hDPSCs cultured at high density. To confirm the inhibitory effect of BAY 11-7085 on NF-κB signaling, we examined p65 phosphorylation in LPS-stimulated high-density hDPSCs. Pretreatment with BAY 11-7085 attenuated LPS-induced p65 phosphorylation, confirming its inhibitory effect on this signaling response under our experimental conditions (Supplementary Figure S2). However, pretreatment with BAY 11-7085, a selective NF-κB inhibitor, had no effect on the expression of osteogenic markers (Figure 5B). These results indicate that NF-κB signaling is not essential for LPS-induced enhancement of osteogenic differentiation in hDPSCs cultured at high density.

### 2.8. Effects of LPS Stimulation on the Proliferation of hDPSCs Cultured at Low and High Density

**Figure 6:**
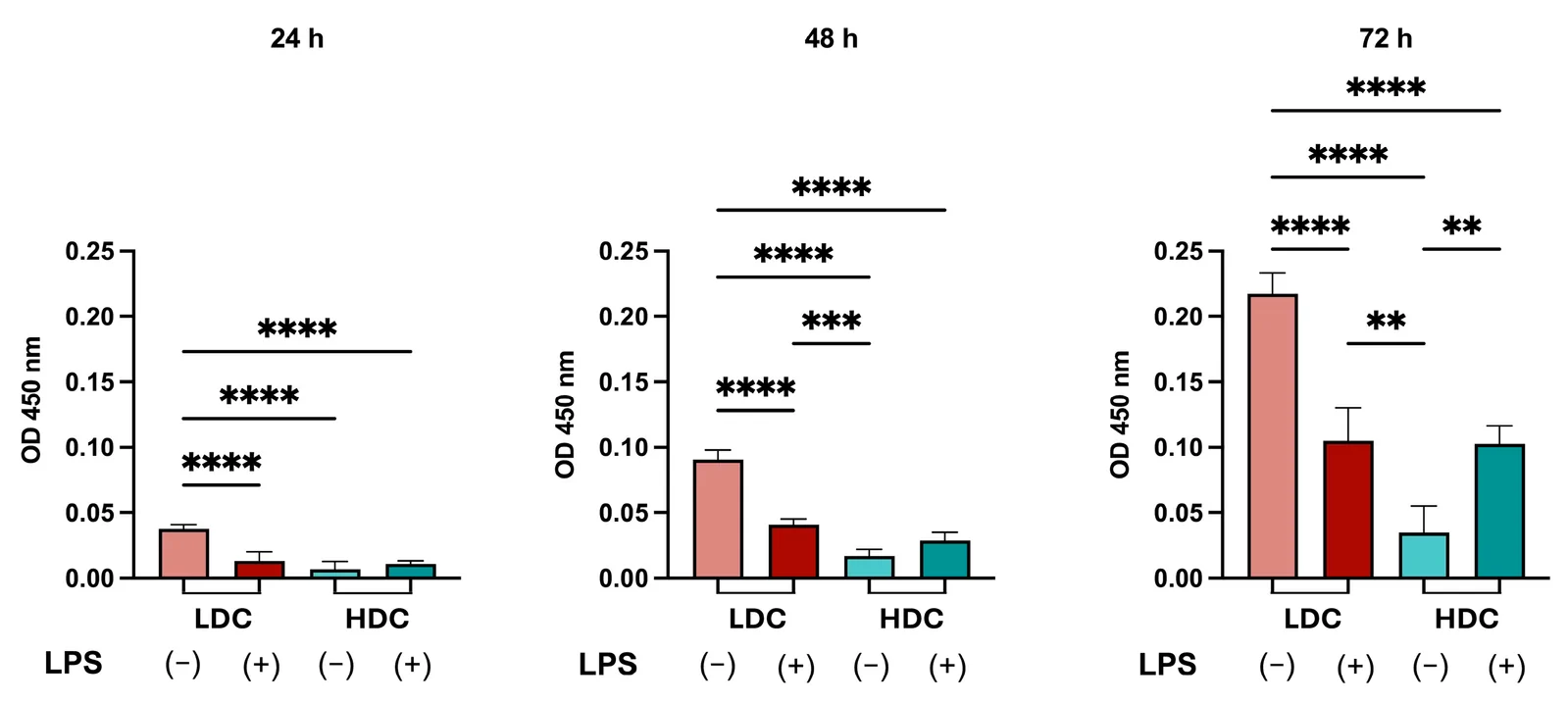
Effects of prior culture density and LPS stimulation on proliferation of hDPSCs. Following 72 h of culture under low- or high-density conditions, hDPSCs were reseeded at the same density for WST-8 assay. LPS stimulation in hDPSCs previously cultured under low-density and high-density culture conditions downregulated the proliferation of hDPSCs. At 72 h, LPS-treated cells previously cultured under high-density conditions exhibited significantly higher WST-8 signal than untreated cells from the same density group. Data are presented as mean ± SD (*n* = 4). ** *p* < 0.01; *** *p* < 0.001; **** *p* < 0.0001. LPS: lipopolysaccharide; hDPSC: human dental pulp stem cell; LDC: low-density culture; HDC: high-density culture.

We assessed the effects of LPS stimulation on the proliferation of hDPSCs cultured at low and high density using the WST-8 assay. Cells previously cultured under high-density conditions significantly reduced cell proliferation. LPS stimulation significantly reduced hDPSCs proliferation under low-density conditions. However, LPS treatment at 72 h significantly increased the hDPSCs’ proliferative capacity compared with high-density culture alone (Figure 6).

## 3. Discussion

In this study, we examined how cell culture density and LPS stimulation interact to regulate the osteogenic differentiation of hDPSCs. We found that high-density culture conditions enhanced osteogenesis-related gene expression, while LPS stimulation further increased *BMP2* and *RUNX2* expression but not *ALPL* or *BGLAP* expression under high-density conditions (Figure 1D). These results were consistent with the heat-map analysis, which showed more pronounced increases in *BMP2* and *RUNX2*, whereas *ALPL* and *BGLAP* exhibited more variable expression patterns (Figure 1C). As osteogenesis-related markers exhibit distinct expression patterns during differentiation [23], these findings suggest that LPS selectively enhances certain osteogenic responses rather than uniformly increasing all osteogenic markers. In contrast, under low-density culture conditions, LPS did not promote the expression of osteogenesis-related genes (Figure 1D). These findings suggest that the cellular microenvironment, potentially involving cell–cell and cell–extracellular matrix interactions that develop under high-density culture, plays a critical role in determining the responsiveness of hDPSCs to LPS.

To elucidate the mechanism by which LPS induces osteogenic differentiation under high-density conditions, we focused on TAK1, a component of the NF-κB signaling pathway. We demonstrated that LPS stimulation markedly increased TAK1 phosphorylation in these cells (Figure 2C). Furthermore, pharmacological inhibition of TAK1 suppressed the expression of osteogenic markers upon LPS stimulation (Figure 3B,C) and reduced mineralized nodule formation (Figure 3D). These results suggest that LPS activates TAK1, which in turn promotes osteogenic differentiation. Notably, LPS-induced TAK1 phosphorylation was below the detection limit in hDPSCs cultured at low density, indicating that both high-density culture and LPS stimulation are required for TAK1 activation.

TAK1 activation in hDPSCs cultured at high density appeared to depend on the cellular context established by integrin–FAK signaling together with LPS stimulation. In line with a report that FAK induces TAK1 activation [24], we found that FAK inhibition suppressed TAK1 phosphorylation in LPS-stimulated hDPSCs at high density (Figure 4A). However, the elevated FAK phosphorylation observed under high-density conditions was not accompanied by a comparable increase in TAK1 phosphorylation in the absence of LPS (Figure 2A,C). These findings suggest that FAK signaling associated with high-density culture may facilitate LPS-induced TAK1 activation, even though LPS does not further increase FAK phosphorylation. Consistent with this interpretation, LPS failed to induce TAK1 phosphorylation and osteogenic differentiation in low-density cultures, where FAK is not activated (Figure 1 and Figure 2A,C). Previous studies have reported both stimulatory [11] and inhibitory [15,16] effects of LPS on osteogenic differentiation, and our findings suggest that integrin signaling may underlie these discrepancies. As high-density culture activates integrin signaling and its downstream PI3K pathway to promote osteogenic differentiation [9], we also examined whether LPS enhances integrin signaling. Although LPS increased phosphorylated p65 expression (Figure 5A), it did not further augment pFAK expression, which was already elevated under high-density culture conditions (Figure 2A). Furthermore, NF-κB inhibition in LPS-stimulated high-density hDPSCs did not reduce FAK phosphorylation (Supplementary Figure S3). These results indicate that LPS-induced NF-κB signaling is unlikely to contribute substantially to the activation of integrin signaling. Although LPS has been reported to induce FAK phosphorylation in Caco-2 intestinal epithelial cells [25] and lung tissue [26], the absence of such an effect in hDPSCs may be explained by cell-type differences or by the possibility that FAK phosphorylation had reached a near-maximal level under high-density culture conditions.

We next investigated how TAK1 contributes to osteogenic differentiation in LPS-stimulated hDPSCs cultured at high density. We first confirmed that phosphorylation of the major MAPKs, ERK, JNK, and p38, was enhanced under high-density culture conditions. Integrin signaling, particularly FAK, has been reported to activate MAPKs [27], and MAPK inhibition suppressed osteogenic differentiation in hDPSCs [18]. Because TAK1 inhibition suppressed LPS-induced phosphorylation of JNK and p38 (Figure 3A), TAK1 likely promotes osteogenic differentiation by further activating MAPK signaling. In addition, transcriptomic analysis revealed significant upregulation of MAPK-associated genes, including *DUSP10*, *JUNB*, and *FOSB*, following LPS stimulation in high-density culture conditions. TAK1, which is a member of MAP3K, regulates bone formation [22,28,29] and mediates the TLR4-dependent activation of JNK and p38 [30]. TAK1-mediated signaling activates JNK through the downstream kinases MKK4 and MKK7, whereas p38 activation is mediated through MKK3 and MKK6 [31]. As TAK1 functions upstream of these signaling cascades, its inhibition is expected to suppress the activation of both JNK and p38. In contrast, ERK is primarily activated through the Ras-Raf-MEK1/2 signaling cascade, in which TAK1 is not directly involved [31]. Consistent with this mechanism, TAK1 inhibition strongly suppressed the phosphorylation of p38 and JNK but had little effect on ERK phosphorylation (Figure 3A), suggesting that TAK1 regulates osteogenic differentiation primarily through JNK and p38 activation. TAK1 is a component of the NF-κB signaling pathway and is activated downstream of LPS/TLR4 stimulation [32,33,34]. However, inhibitors of IRAK4 and TRAF6, which are key components of the canonical NF-κB pathway, did not suppress MAPK phosphorylation in LPS-stimulated high-density hDPSCs (Supplementary Figure S4). These findings suggest that, in hDPSCs cultured at high density, LPS may induce TAK1 phosphorylation through a mechanism largely independent of canonical NF-κB signaling. This interpretation is further supported by the observation that IRAK4 and TRAF6 inhibitors failed to inhibit LPS-induced TAK1 phosphorylation under high-density conditions (Figure 4B). Thus, our findings raise the possibility that TRAF6-independent mechanisms contribute to TAK1 phosphorylation in high-density hDPSCs. One potential mechanism is activation of the TRIF/Pellino axis [35], which is reported to promote Pellino-mediated TAK1 phosphorylation and subsequent MAPK activation [36,37].

The growth suppression observed in hDPSCs previously cultured under high-density culture conditions is consistent with previous reports showing that increased cell density promotes cell-cycle arrest and drives stem cells toward lineage commitment [9,38]. Under high-density conditions, enhanced cell–cell contact and extracellular matrix deposition may activate mechanotransduction pathways that promote differentiation while simultaneously suppressing proliferation [9,39]. In hDPSCs previously cultured at low density, LPS stimulation reduced proliferative activity, likely due to LPS-induced inhibition of cell proliferation [40] or enhanced apoptosis [41]. Notably, the proliferation suppressed under prior high-density conditions showed a significant increase in proliferation following LPS stimulation at 72 h, whereas this increase was attenuated by TAK1 inhibition with NG25 (Supplementary Figure S5). TAK1 regulates the expression and activity of numerous cell-cycle-related genes, and inhibition of TAK1 suppresses the proliferation of rhabdomyosarcoma cells [42].

Various concentrations of LPS have been used in previous studies, depending on the degree of inflammatory stimulation required [43]. In hDPSCs, LPS at concentrations of 100 and 500 ng/mL has been reported to promote osteo/odontogenic differentiation without affecting cell proliferation during a 3-day observation period [11]. In addition, 100 ng/mL LPS elicits robust cytokine and chemokine expression in human dental pulp cells [44,45]. Based on these findings, we selected 100 ng/mL LPS as a lower effective concentration to induce an inflammatory environment in this study. Clinically, even after careful removal of necrotic or infected tissue, bacterial metabolites such as LPS may remain in the local environment. Therefore, our experimental conditions were designed to model aspects of clinically relevant inflammatory settings and allow us to evaluate how bacterial components influence the osteogenic potential of hDPSCs. This study provides mechanistic insights into the interplay between cell density, inflammatory signaling, and osteogenic differentiation in hDPSCs. However, several limitations of this work should be acknowledged. First, all experiments were conducted in vitro and may not have fully recapitulated the complexity of the in vivo microenvironment. Second, our mechanistic conclusions rely primarily on pharmacological inhibitors; genetic approaches will be necessary to further validate pathway specificity. Future studies should elucidate the molecular mechanisms governing integrin–TAK1 crosstalk and evaluate the functional significance of this signaling axis in physiologically relevant animal models.

Our findings reveal a previously underappreciated link between integrin-mediated mechanotransduction and inflammatory signaling in hDPSCs. By demonstrating that the osteogenic effects of inflammatory stimuli depend, at least in part, on the cellular microenvironment, this study provides valuable insights that may contribute to the development of improved strategies for dental and bone tissue engineering under inflammatory conditions.

## 4. Materials and Methods

### 4.1. Isolation of hDPSCs

This study was conducted in accordance with the Declaration of Helsinki and approved by the Ethical Committee of Tokyo Medical and Dental University (now Institute of Science Tokyo, Tokyo, Japan; D2023-066). Informed consent was obtained from all participants. The hDPSCs used in this study were previously isolated from freshly extracted human third molars and characterized by the expression of mesenchymal stem cell markers (CD44, CD73, CD90, CD105, and CD146), absence of the hematopoietic cell marker CD34, and multilineage differentiation potential, as reported in our previous studies [9,46]. In addition, flow cytometric analysis performed in the present study demonstrated negligible expression of CD31 (0.21%) and CD45 (0.33%), further supporting the absence of endothelial and hematopoietic cell contamination in the hDPSC population (Supplementary Figure S6). The cells were cultured in alpha-modified minimum essential medium (α-MEM; Fujifilm Wako Pure Chemical, Osaka, Japan) supplemented with 10% fetal bovine serum (FBS; Thermo Fisher Scientific, Waltham, MA, USA) and 1% penicillin/streptomycin (Fujifilm Wako) under standard conditions (37 °C, 5% CO_2_). Cells at passages 3 to 5 were used for the experiments.

### 4.2. Cell Culture Conditions and Experimental Groups

hDPSCs were seeded under low-density (5 × 10^3^ cells/cm^2^) or high-density (1 × 10^5^ cells/cm^2^) conditions. These seeding densities were selected based on our previous study, which demonstrated that hDPSCs seeded at 5 × 10^3^ cells/cm^2^ remained sparsely distributed for 3 days, whereas those seeded at 1 × 10^5^ cells/cm^2^ formed a confluent, densely packed culture [9].

For the low-density condition, 3 × 10^5^ cells were seeded in 100 mm culture dishes, whereas for the high-density condition, 4 × 10^5^ cells were seeded in 12-well plates. Apart from the seeding density and culture vessel, all groups were maintained under the same culture conditions, including culture medium, incubation conditions, treatment schedule, and culture duration. Cells assigned to the LPS-treated groups were stimulated with 100 ng/mL LPS (*Escherichia coli* O111:B4; Sigma-Aldrich, St. Louis, MO, USA) at the indicated time points. Cells cultured under low-density conditions with LPS were designated as the LPS-LDC group, whereas those cultured under high-density conditions with LPS were designated as the LPS-HDC group. The corresponding untreated low- and high-density cultures served as the LDC and HDC control groups, respectively. All experimental groups were maintained under the same serum-containing culture conditions for 3 days before RNA and protein extraction.

### 4.3. RNA Extraction and RT-qPCR

Total RNA (150 ng) extracted from hDPSCs using the QuickGene-Mini80 system (Fujifilm Wako) was reverse-transcribed into cDNA using PrimeScript™ RT Master Mix (Takara Bio, Shiga, Japan). RT-qPCR was performed using the synthesized cDNA, GoTaq qPCR MasterMix (Promega, Madison, WI, USA), and specific primers (Table 1) on a CFX96 Real-Time qPCR System (Bio-Rad, Kidlington, UK). *ACTB* was used as an internal control.

### 4.4. RNA-Sequencing Analysis

The prepared RNA samples were sequenced on an Illumina DNBSEQ platform by Novogene Co., LTD (Beijing, China) through Filgen, Inc (Nagoya, Japan). Differential expression analysis was conducted using DESeq2. Principal component analysis, volcano plot and heatmap visualization were performed in R software (version 4.5.3; R Foundation for Statistical Computing, Vienna, Austria). Gene ontology and KEGG enrichment analyses were conducted to investigate the biological functions associated with the differentially expressed genes.

### 4.5. Western Blotting

hDPSCs (4 × 10^5^ cells) were lysed in 100 μL of radioimmunoprecipitation buffer containing a phosphatase inhibition cocktail (PhosSTOP; Sigma-Aldrich, St. Louis, MO, USA) and a protease inhibition cocktail (cOmplete; Sigma-Aldrich). Lysate samples were separated by 10% SDS-PAGE and transferred onto polyvinylidene difluoride membranes (Immobilon-P; Merck Millipore, Burlington, MA, USA) using a semidry transfer system (0.15 mA, 1 h, WSE-4040; ATTO, Tokyo, Japan). After blocking, the blots were incubated overnight at 4°C with the following primary antibodies: anti-TAK1 (1:1000, D948D7, #5206, monoclonal, rabbit; Cell Signaling Technology, Danvers, MA, USA), anti-phospho-TAK1 (1:1000, Thr184/187, 90C7, #4508, monoclonal, rabbit; Cell Signaling Technology), anti-FAK (1:1000, D507U, #71433, monoclonal, rabbit; Cell Signaling Technology), anti-phospho-FAK (1:1000, Tyr397, #3283, polyclonal, rabbit; Cell Signaling Technology), anti-p44/42 MAPK (Erk1/2) (1:1000, 137F5, #4695, monoclonal, rabbit; Cell Signaling Technology), anti-phospho-p44/42 MAPK (Erk1/2) (1:1000, Thr202/Tyr204, D13.14.4E, #4370, monoclonal, rabbit; Cell Signaling Technology), anti-p38 (1:1000, D13E1, #8690, monoclonal, rabbit; Cell Signaling Technology), anti-phospho-p38 (1:1000, Thr180/Tyr182, D3F9, monoclonal, rabbit; Cell Signaling Technology), anti-stress-activated protein kinase/c-Jun N-terminal kinase (SAPK/JNK) (1:1000, #9252, polyclonal, rabbit; Cell Signaling Technology), anti-phospho-SAPK/JNK (1:1000, Thr183/Tyr185, 81E11, #4668, monoclonal, rabbit; Cell Signaling Technology), anti-NF-κB p65 (1:1000, D14E12, #8242, monoclonal, rabbit; Cell Signaling Technology), anti-phospho-NF-κB p65 (1:1000, Ser536, 93H1, #3033, monoclonal, rabbit; Cell Signaling Technology), and anti-GAPDH (1:4000, PM053-7; Medical & Biological Laboratories, Nagoya, Japan). Horseradish peroxidase (HRP)-conjugated anti-rabbit IgG (1:10,000, W4011; Promega) was used as secondary antibody. Protein bands were visualized using a chemiluminescent HRP substrate (Immobilon, Millipore), and images were captured with a LAS-3000 mini-imaging system (Fujifilm Wako). Band intensities were quantified using ImageJ software (ver. 1.53t; National Institutes of Health, Bethesda, MD, USA), and expression levels were calculated based on integrated pixel density. For ERK1/2 and JNK, the intensities of both bands were summed to obtain the total band intensity for densitometric quantification.

### 4.6. Osteogenic Differentiation

hDPSCs (4 × 10^4^ cells/well) were seeded in a 48-well plate and cultured in α-MEM containing 10% FBS. After 24 h, the medium was replaced with osteogenic inducing medium containing β-glycerophosphate (5 mM; Fujifilm Wako), L-ascorbic acid (0.2 mM; Fujifilm Wako), and dexamethasone (1 nM; Fujifilm Wako) with or without LPS. The medium was changed every 2 days. Mineralized nodule formation was assayed by Alizarin Red S (Fujifilm Wako) staining following methanol fixation. Images were acquired and staining intensity was quantified using ImageJ software.

### 4.7. Cell Proliferation

hDPSCs were prepared in four experimental groups. After 3 days of culture, cells were seeded in 96-well plates (3 × 10^3^ cells/well) and cultured for 24, 48, and 72 h. In the LPS-stimulated groups, LPS treatment was continued throughout the assay period. Cell proliferation was evaluated using the WST-8 assay (Cell Counting Kit-8; Dojindo Molecular Technologies, Kumamoto, Japan). After 1 h of incubation with the reagent, the absorbance at 450 nm was measured using a microplate reader (Sunrise; Tecan, Männedorf, Switzerland).

### 4.8. Inhibitors

NG25 (5 μM) and HS-276 (4 μM) (TAK1 inhibitors; Selleck Chemicals, Houston, TX, USA), IRAK4-IN-1 (10 μM) (IRAK4 inhibitor; Selleck Chemicals), C25-140 (20 μM) (TRAF6 inhibitor; Selleck Chemicals), FAK inhibitor 14 (1 μM) (FAK inhibitor; Cayman Chemical, Ann Arbor, MI, USA), and BAY 11-7085 (1 μM) (NF-κB inhibitor, 196309-76-9; Cayman Chemical) were dissolved in dimethyl sulfoxide and applied to hDPSCs 1 h before LPS stimulation.

### 4.9. Statistical Analysis

Statistical analysis was conducted using GraphPad Prism 9.0 (GraphPad Software, San Diego, CA, USA). One-way analysis of variance (ANOVA) followed by Tukey’s post hoc test and two-way ANOVA followed by Šídák’s and Dunnett’s tests were used for comparisons among multiple groups, and Student’s *t*-test was used for two groups. A *p* value < 0.05 was considered statistically significant. All experiments were performed at least in triplicate.

## 5. Conclusions

High-density culture conditions increased FAK phosphorylation and enhanced osteogenesis-related responses in hDPSCs. LPS stimulation further increased *BMP2* and *RUNX2* mRNA expression and mineralized nodule formation under high-density conditions. Mechanistically, LPS stimulation induced TAK1 phosphorylation and downstream activation of p38 and JNK, while TAK1 inhibition attenuated these signaling responses and osteogenic outcomes. Furthermore, FAK inhibition attenuated TAK1 activation in LPS-stimulated high-density hDPSCs. These findings suggest that the enhanced osteogenic responses observed under the combination of high-density culture and LPS stimulation involve an interaction between FAK-associated signaling and the TAK1–MAPK pathway.

## Figures and Tables

**Table 1:** Primer sequences.

Gene	Forward	Reverse	Accession No.	Size (bp)
*ACTB*	5′-CTGACTGACTACCTCATGAAGATCC-3′	5′-GTAGCACAGCTTCTCCTTAATGTCA-3′	NM_001101	102
*RUNX2*	5′-CCTCATCCCAGTATGAGAGTAGGTG-3′	5′-CTGGGGTCTGTAATCTGACTCTGTC-3′	NM_001278478	110
*ALPL*	5′-ATGCTGAGTGACACAGACAAGAAG-3′	5′-GGTAGTTGTTGTGAGCATAGTCCAC-3′	NM_000478	124
*BGLAP*	5′-CCTTTGTGTCCAAGCAGGAG-3′	5′-TCAGCCAACTCGTCACAGTC-3′	NM_199173	151
*BMP2*	5′-GACGTTGGTCAACTCTGTTAACTCT-3′	5′-CACAACCATGTCCTGATAGTTCTTT-3′	NM_001200	133

Abbreviations: *ACTB*: actin beta; *RUNX2*: runt-related transcription factor 2; *ALPL*: alkaline phosphatase, biomineralization associated; *BGLAP*: bone gamma-carboxyglutamate protein; *BMP2*: bone morphogenetic protein 2.

## Data Availability

The original contributions presented in this study are present in the main text of the manuscript and Supplementary Materials. Further inquiries are available from the corresponding author upon reasonable request.

## Supplementary Materials

The following supporting information can be downloaded at: https://www.mdpi.com/article/10.3390/ijms27219515/s1
