# Supplementary material for: High-Density Culture and Lipopolysaccharide Cooperatively Activate TAK1–MAPK Signaling to Promote Osteogenic Differentiation in Human Dental Pulp Stem Cells
Source: Int J Mol Sci. 2026 Oct 24;27(21):9515. doi: 10.3390/ijms27219515 (PMC13763265; doi:10.3390/ijms27219515)
Supplement: Supplementary file 1 [file ijms-27-09515-s001.zip › Full Length WB images-20261020.pdf]

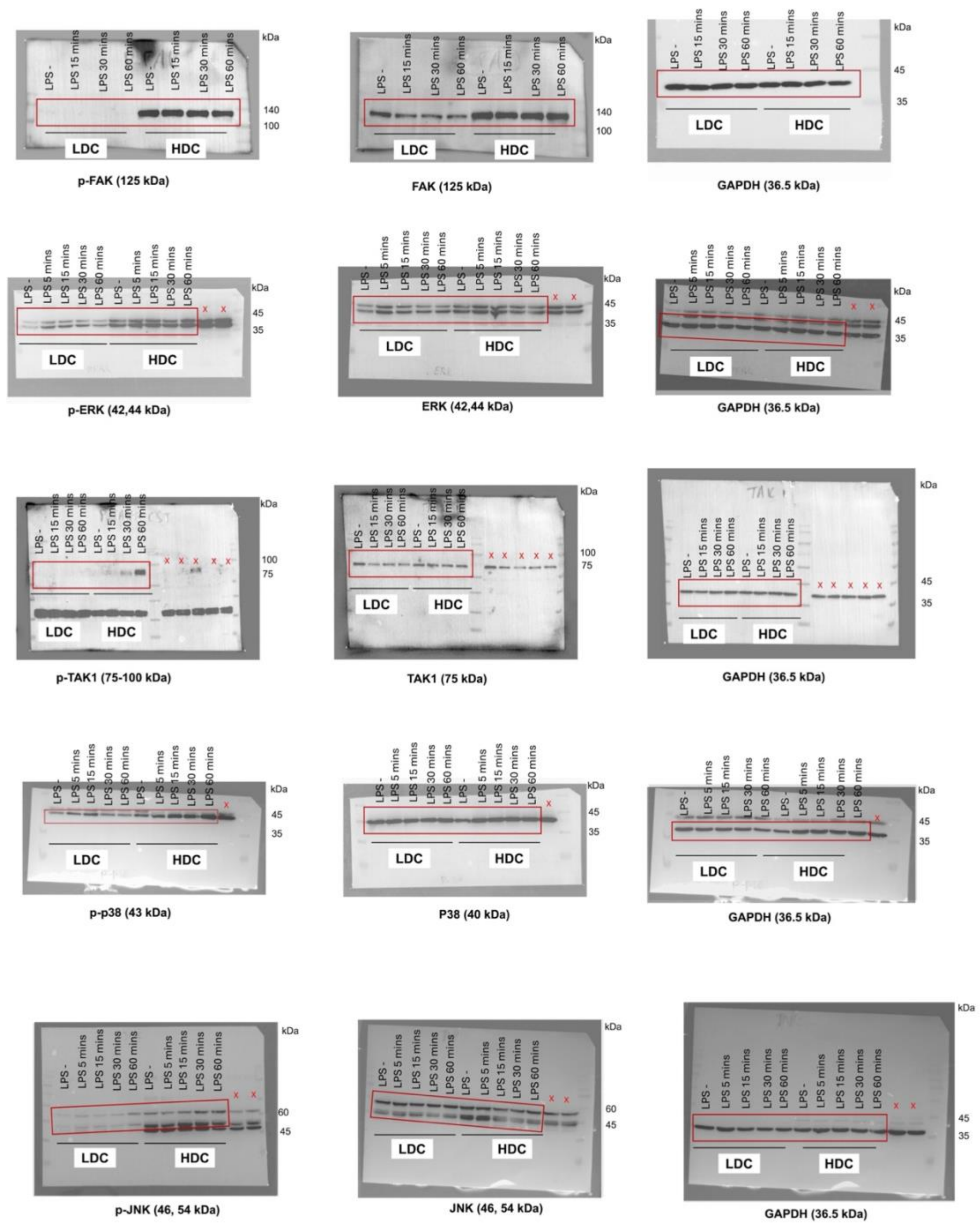

Full length western blot images for Figure 2. Each GAPDH blot corresponds to the blots in the same row.

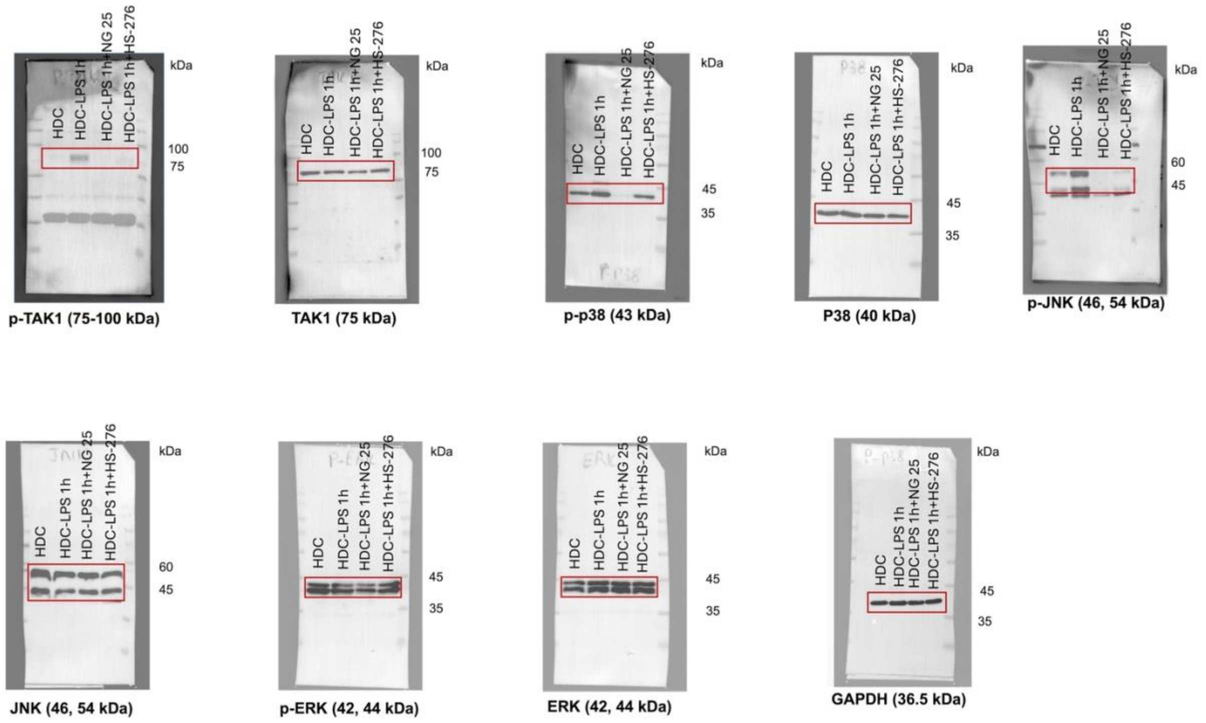

Full length western blot images for Figure 3. GAPDH loading control corresponds to all protein blots presented in the figure.

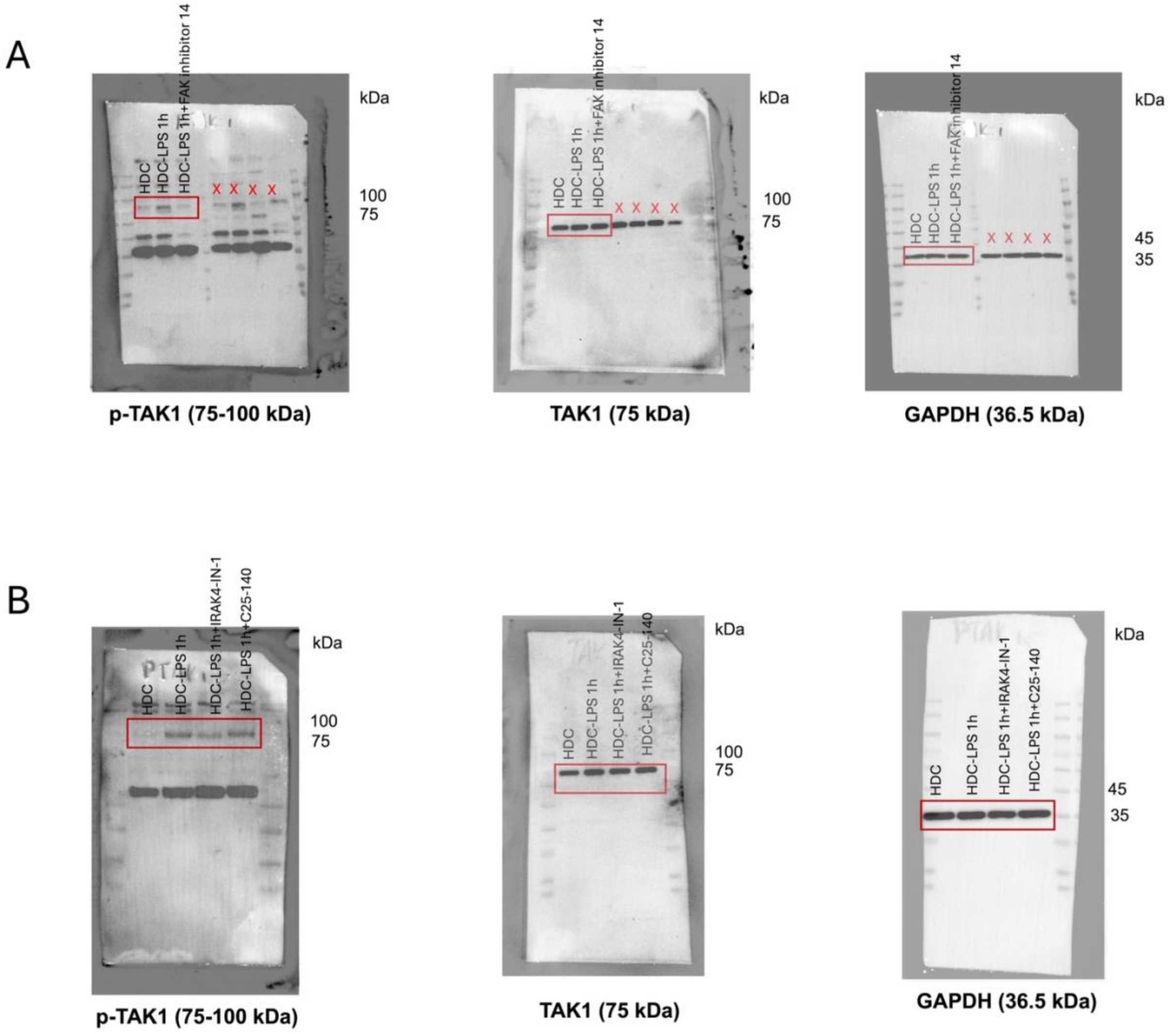

Full length western blot images for Figure 4A and 4B. Each GAPDH blot corresponds to the blots in the same row.

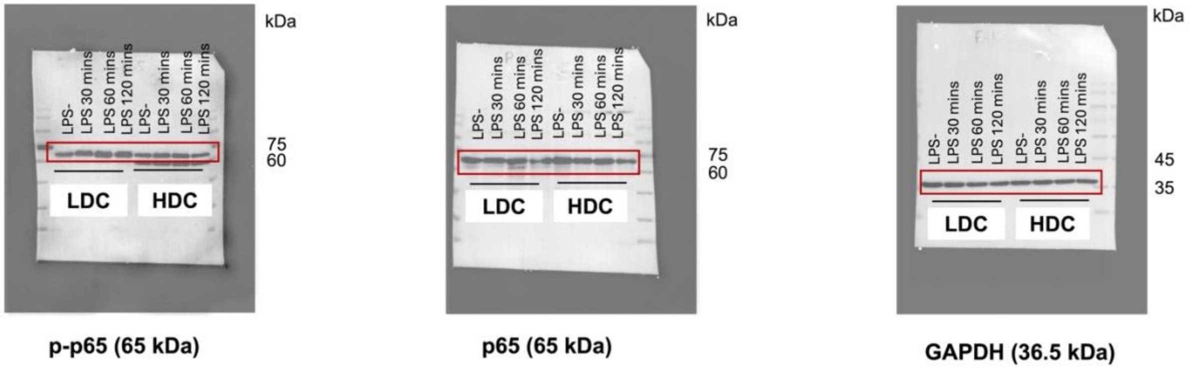

Full length western blot images for Figure 5. GAPDH blot corresponds to the blots in the same row.

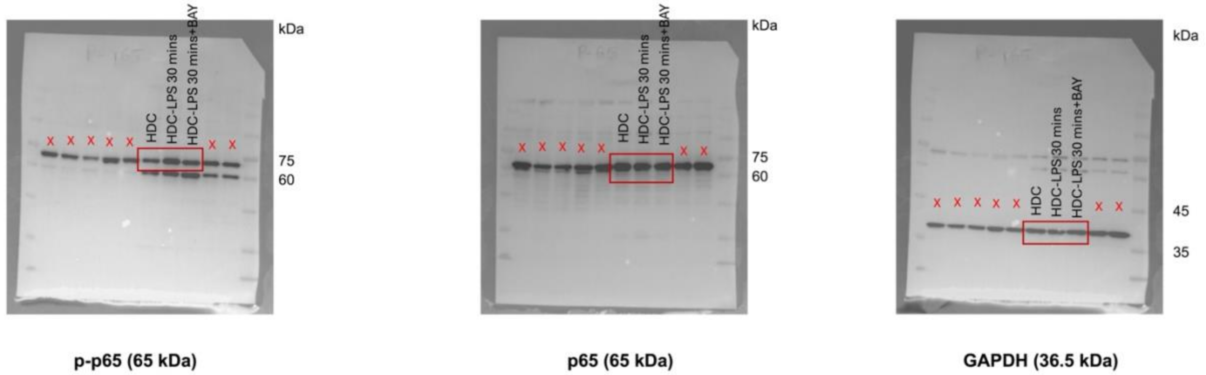

Full length western blot images for Supplementary Figure 2. GAPDH blot corresponds to the blots in the same row.

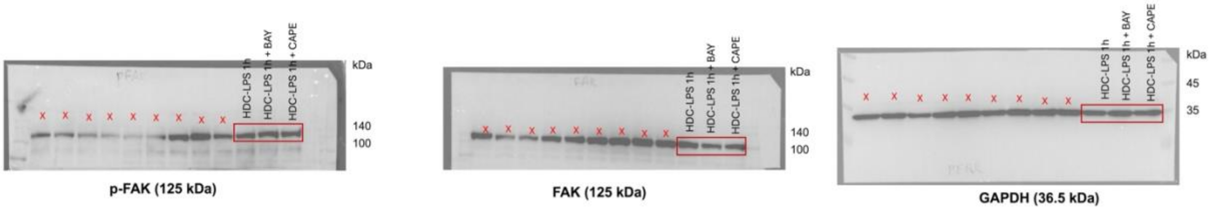

Full length western blot images for Supplementary Figure 3. GAPDH blot corresponds to the blots in the same row.

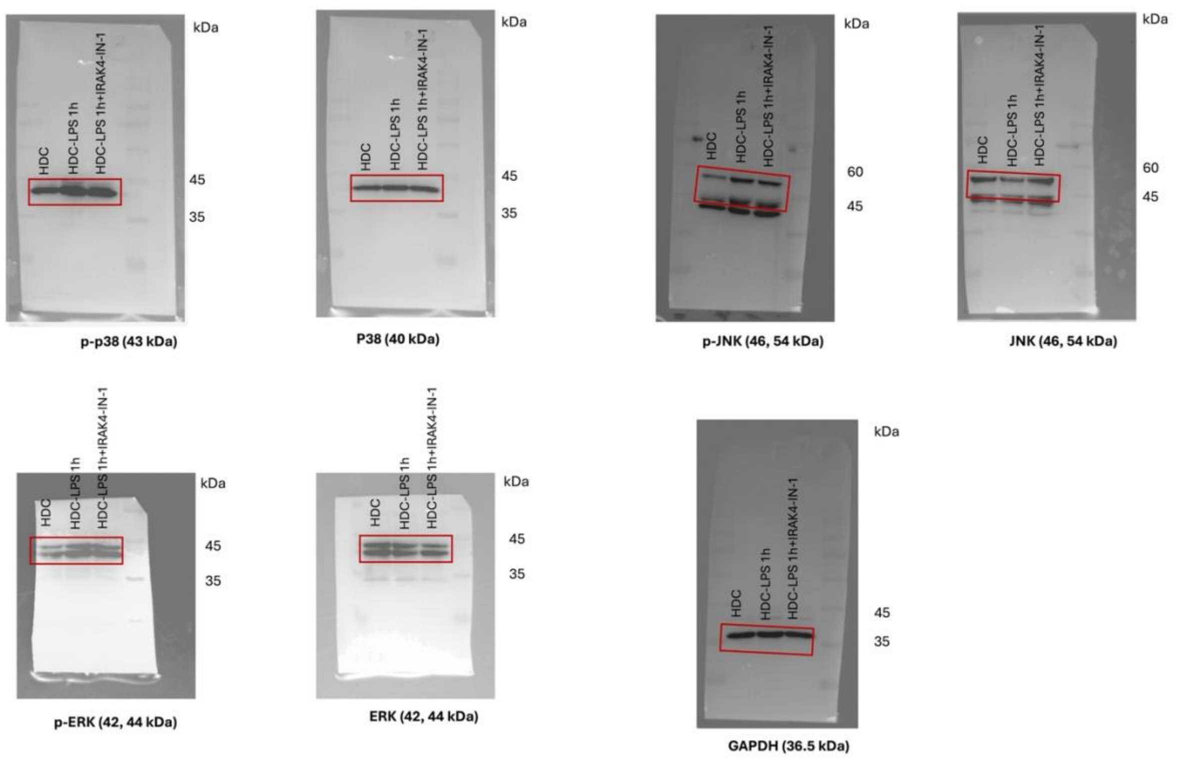

Full length western blot images for Supplementary Figure 4A. GAPDH loading control corresponds to all protein blots presented in the figure.

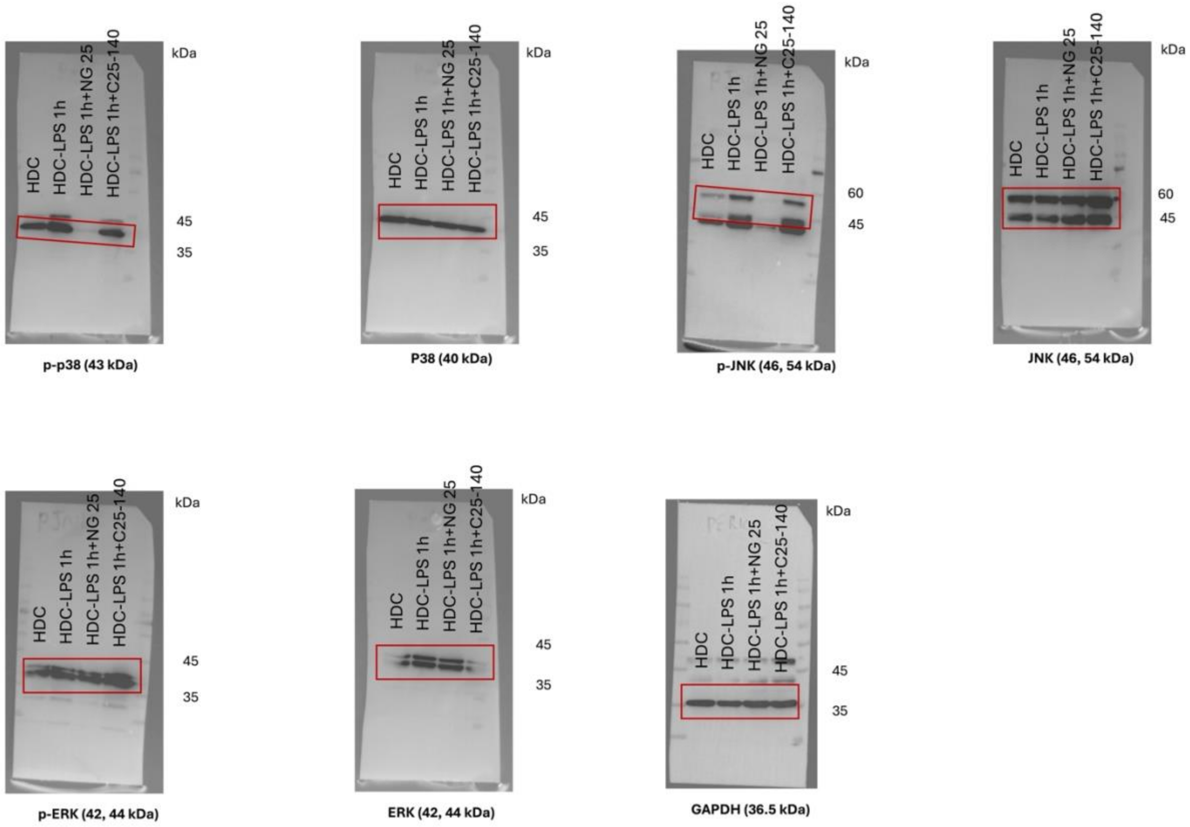

Full length western blot images for Supplementary Figure 4B. GAPDH loading control corresponds to all protein blots presented in the figure.
