# Supplementary material for: High-Density Culture and Lipopolysaccharide Cooperatively Activate TAK1–MAPK Signaling to Promote Osteogenic Differentiation in Human Dental Pulp Stem Cells
Source: Int J Mol Sci. 2026 Oct 24;27(21):9515. doi: 10.3390/ijms27219515 (PMC13763265; doi:10.3390/ijms27219515)
Supplement: Supplementary file 1 [file ijms-27-09515-s001.zip › Supplementary material-20261020.pdf]

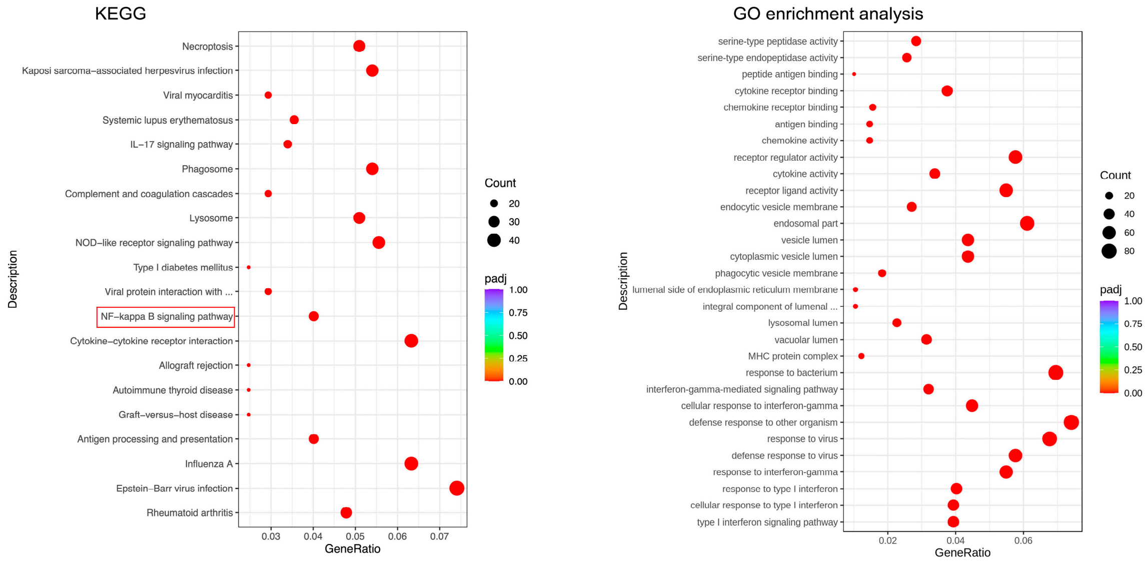

Supplementary Figure S1. Functional enrichment analysis of upregulated genes in LPS-stimulated high-density hDPSCs.

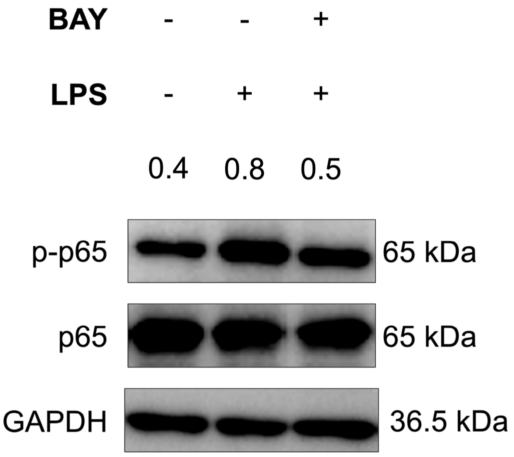

Supplementary Figure S2. Effect of BAY 11-7085 on LPS-induced p65 phosphorylation in high-density cultured hDPSCs. LPS-induced upregulation of p65 phosphorylation was attenuated by pretreatment with BAY 11-7085 (5 μM) for 1 h in hDPSCs cultured under high-density conditions. LPS: lipopolysaccharide; hDPSC: human dental pulp stem cell; BAY: BAY 11-7085.

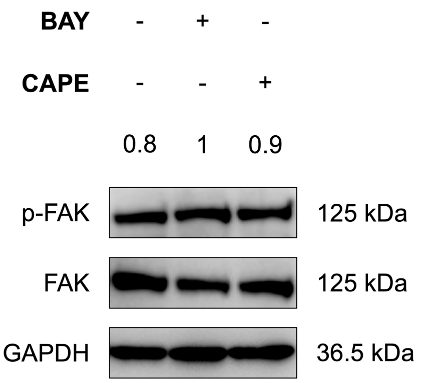

Supplementary Figure S3. Effect of NF-κB inhibitors on FAK phosphorylation. Neither of NF-κB inhibitors (5 μM BAY 11-7085 and 5 μM CAPE) blocked FAK phosphorylation in LPS-stimulated high-density hDPSCs. LPS: lipopolysaccharide; hDPSC: human dental pulp stem cell; BAY: BAY 11-7085; CAPE: caffeic acid phenethyl ester.

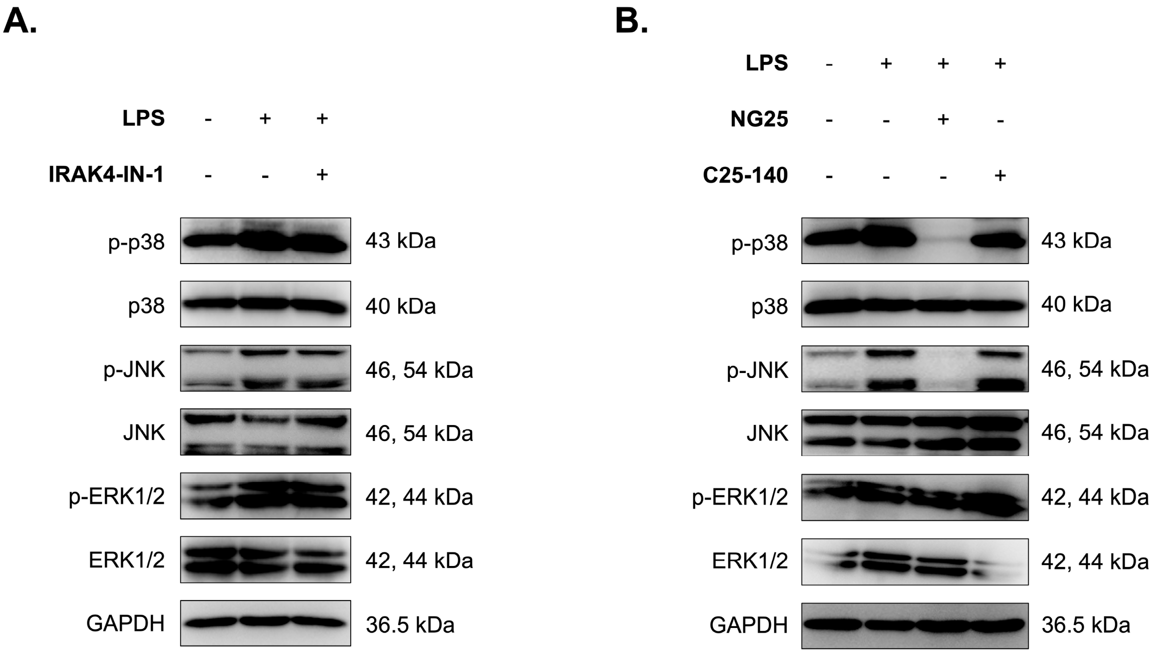

Supplementary Figure S4. Effects of IRAK4, TRAF6, and TAK1 inhibitors on MAPK phosphorylation. Neither the IRAK4 inhibitor (10 μM IRAK4-IN-1: **A**) nor the TRAF6 inhibitor (20 μM C25-140: **B**) suppressed MAPK phosphorylation in LPS-stimulated hDPSCs cultured at high density. In contrast, TAK1 inhibitor (5 μM NG25) robustly suppressed MAPK phosphorylation under the same conditions. LPS: lipopolysaccharide; hDPSC: human dental pulp stem cell.

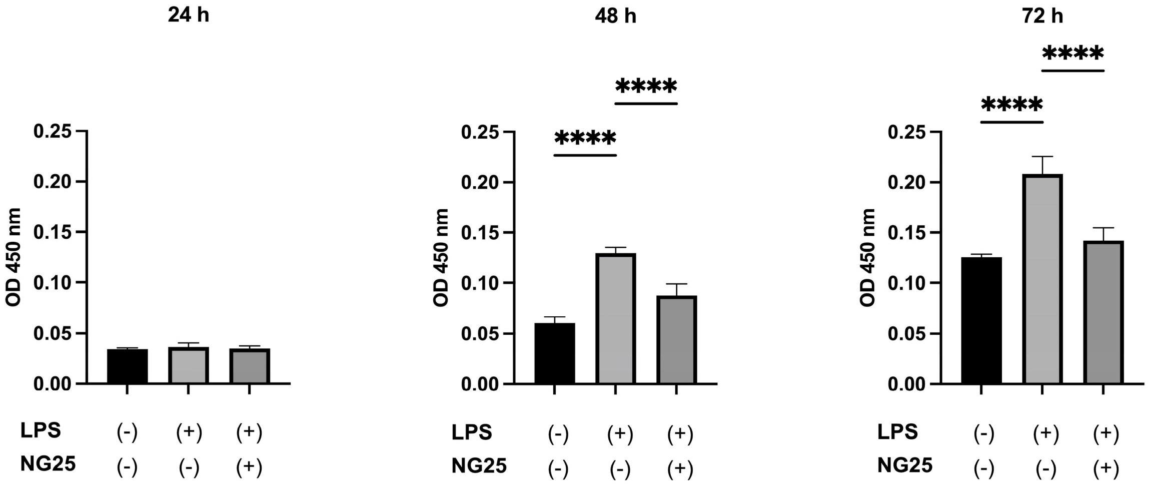

Supplementary Figure S5. Effect of TAK1 inhibition on the viability of hDPSCs, as determined by WST-8 assay. TAK1 inhibitor (5 μM NG25) significantly downregulated proliferation of LPS-stimulated high-density hDPSCs. The error bar represents SD (n=5). **** $p < 0.0001$. hDPSC: human dental pulp stem cell; LPS: lipopolysaccharide.

Flow Cytometric Analysis of CD31 and CD45 Markers

Human dental pulp stem cells (hDPSCs) were harvested and washed with FACS buffer consisting of phosphate-buffered saline supplemented with 2% fetal bovine serum. A total of 5×10^5 cells were resuspended in 50 μL of FACS buffer and incubated with PE-conjugated mouse anti-human CD31 antibody (clone WM59, #555446, BD Biosciences, San Jose, CA, USA) or BV510-conjugated mouse anti-human CD45 antibody (clone HI30, #563204, BD Biosciences) for 30 min on ice in the dark. Corresponding PE- and BV510-conjugated isotype antibodies were used as negative controls, and an unstained cell sample was included to assess cellular autofluorescence. After antibody staining, the cells were washed with FACS buffer, resuspended in FACS buffer, and analyzed using a flow cytometer (BD FACSLyric; BD Biosciences).

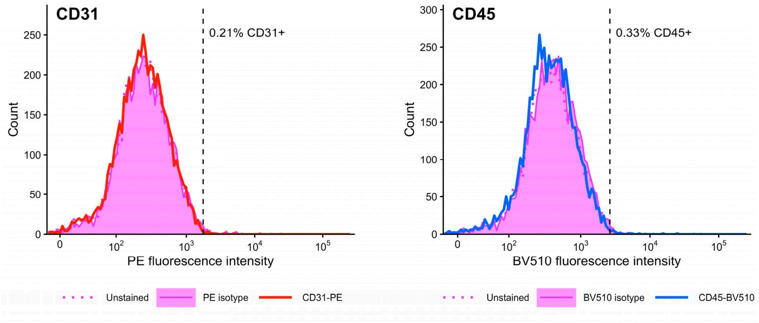

Supplementary Figure S6. Flow cytometric analysis of CD31 and CD45 expression in hDPSCs. Overlay histograms show CD31-PE and CD45-BV510 fluorescence. Unstained cells and the corresponding isotype controls were used as negative controls. hDPSC: human dental pulp stem cell.
